# Single-Port Robotic-Assisted Pneumovesicoscopic Cohen Ureteral Reimplantation for Persistent High-Grade Bilateral Vesicoureteral Reflux in a Child: A Case Report

**DOI:** 10.3390/children13091205

**Published:** 2026-09-07

**Authors:** Bo-Yang Shie, Wei-Hsiang Su, Chia-Man Chou, Sheng-Yang Huang

**Affiliations:** 1 Division of Pediatric Surgery, Department of Surgery, Taichung Veterans General Hospital, Taichung City 407219, Taiwan; boyangshie@vghtc.gov.tw (B.-Y.S.); william820511@vghtc.gov.tw (W.-H.S.); cmchou@vghtc.gov.tw (C.-M.C.); 2 School of Medicine, National Yang Ming Chiao Tung University, Taipei City 112304, Taiwan; 3 Department of Post-Baccalaureate Medicine, College of Medicine, National Chung Hsing University, Taichung City 402202, Taiwan

**Keywords:** vesicoureteral reflux, Cohen ureteral reimplantation, pneumovesicoscopy, single-port robotic surgery, case report

## Abstract

**Highlights:**

**What are the main findings?**

- To our knowledge, this is the first clinical report of pure intravesical bilateral Cohen ureteral reimplantation using the da Vinci SP system in a child.
- The procedure was completed without conversion or intraoperative complications; at six months, the patient was asymptomatic, with reassuring urinalysis and no hydronephrosis or obstruction on ultrasonography.

**What is the implication of the main finding?**

- Single-port robotic articulation may facilitate complex bilateral pneumovesicoscopic reconstruction through a single transumbilical bladder access in carefully selected children.
- As a single case without postoperative reflux-specific imaging, this report establishes technical feasibility only; comparative multicenter studies with long-term clinical, imaging, and renal functional follow-up are required.

**Abstract:**

Background/Objectives: High-grade vesicoureteral reflux (VUR) may require definitive reconstruction when conservative or endoscopic management fails. Although robot-assisted ureteral reimplantation is increasingly used in children, published experience has predominantly involved an extravesical approach or multiport intravesical surgery. We describe a pure intravesical Cohen reimplantation performed with the da Vinci Single Port (SP) system. Case Presentation: A 7-year-old girl had recurrent febrile urinary tract infections and acute pyelonephritis despite two previous dextranomer/hyaluronic acid copolymer injections. Voiding cystourethrography demonstrated persistent bilateral VUR (right grade V and left grade IV). Technetium-99m dimercaptosuccinic acid renal scintigraphy showed differential renal function of 38.98% on the right and 61.02% on the left, with cortical defects consistent with renal scarring. She underwent single-port robot-assisted pneumovesicoscopic bilateral Cohen ureteral reimplantation. The previous injectable material was removed, bilateral cross-trigonal submucosal tunnels were created, and both ureters were reimplanted with interrupted 5-0 polyglactin sutures. The total anesthesia duration, operative duration and console duration was 375 min, 310 min and 250 min, respectively. There was minimal blood loss and no drainage tubes or Double J stents were placed. No intraoperative complication occurred. A transient episode of emesis with stool impaction on postoperative day 2 resolved after a glycerin enema and brief bowel rest. She tolerated a regular diet by postoperative day 4 and was discharged on postoperative day 5. At early 6-month follow-up, the patient remained symptom free and ultrasonography showed no obstruction. Conclusions: This case demonstrates the technical feasibility of a pure intravesical da Vinci SP Cohen reimplantation in a child with refractory bilateral high-grade VUR. Comparative effectiveness of different approaches and longer clinical follow-up are required in the future.

## 1. Introduction

Open ureteral reimplantation remains a benchmark treatment for pediatric vesicoureteral reflux (VUR). Pneumovesicoscopy reproduces the intravesical Cohen cross-trigonal repair through a minimally invasive approach and has achieved favorable outcomes [1,2,3,4,5]. Compared with open surgery, it may shorten hospitalization and reduce perioperative morbidity, but usually requires longer operating time and advanced intracorporeal suturing [2,3]. Intravesical Politano-Leadbetter reconstruction has also been described but may be more technically demanding [6,7].

Robotic assistance provides magnified three-dimensional vision, wristed instruments, and improved suturing ergonomics; however, most pediatric robot-assisted reimplantations use the extravesical Lich-Gregoir approach [8]. Intravesical robotic Cohen reimplantation has been reported in an animal model and limited multiport clinical experience [9,10,11,12]. Pediatric da Vinci Single Port (SP) applications remain selective [13], and reduced-port ureteral reimplantation has mainly used single-port-plus-one extravesical surgery [14,15]. A 2026 animal study described single-port pneumovesical Cohen reconstruction using another robotic platform [16]. To our knowledge, no previous clinical report has described a pure intravesical pneumovesicoscopic Cohen reimplantation using the da Vinci SP system in a child. We reported this case with detailed technical proof of concept and early postoperative course.

## 2. Case Presentation

### 2.1. Patient Information and Symptoms

The patient was born in 2018 at approximately 38 + 2 weeks, with a birth weight of 3430 g. The prenatal and neonatal periods were described as unremarkable. Subsequent growth and developmental milestones were normal, and routine vaccinations were received according to the national immunization schedule. By infancy, she had bilateral VUR, initially Right Grade IV and Left Grade V, associated with breakthrough urinary tract infection.

On 1 May 2019, because of breakthrough urinary tract infection (UTI), she underwent the first bilateral subureteral Deflux injection. Cystoscopy showed an abnormal right ureteral orifice and a laterally positioned, “golf-hole” appearing left ureteral orifice, with mild bladder trabeculation. Deflux, 1.0 mL per ureteral orifice, was injected. After this procedure, subsequent study revealed residual reflux to Right Grade II and Left Grade IV.

Because significant residual reflux persisted, she underwent a second bilateral Deflux injection on 20 August 2019. Both ureteral orifices were observed as loose; on the left, there was peripheral muscle weakness and migration of the previous Deflux material. Another 1.0 mL of Deflux was injected into each ureteral orifice.

She remained under regular outpatient follow-up without episodes of UTI Before the 2023 hospitalization. Renal ultrasound showed generally normal bilateral renal appearance, although the right kidney was approximately 1 cm smaller than the left. She was not receiving regular antibiotic prophylaxis at that time.

At age 4, she developed approximately four days of fever, reaching 40 °C, with decreased appetite and activity. Emergency evaluation showed elevated inflammatory markers and mild pyuria. She was admitted with a working diagnosis of febrile UTI in the setting of bilateral VUR. Initial laboratory data included CRP 12.3 mg/dL, creatinine 0.47 mg/dL, and urinalysis with 6–9 WBC/HPF, trace leukocyte esterase, and negative nitrite. She initially received cefepime from 21–22 April 2023, followed by cefazolin plus gentamicin from 22–24 April, together with IV hydration. Fever resolved by the night of 23 April. A DMSA renal scan and abdominal sonography were arranged on 24 April for renal follow-up, without significant change comparing with the previous studies. She was discharged in stable condition.

In July 2024, she again developed high fever and decreased appetite. Fever reached approximately 39.8 °C in the emergency department. Laboratory studies showed WBC 15,220/µL, CRP 6.0 mg/dL, and pyuria. She was admitted with complicated UTI associated with VUR. She was treated with cefazolin plus gentamicin from 7–11 July. During the admission: Kidney sonography on 8 July 2024 showed mild hydronephrosis of the right renal pelvis. Uroflowmetry/bladder scan on 10 July revealed maximum flow 29.2 mL/s, average flow 16.5 mL/s, voided volume 178 mL, and post-void residual 29 mL. The interpretation was good uroflow with scant residual urine. She became afebrile with improved activity and appetite and was discharged on 11 July. Bladder/bowel and voiding-habit modification was emphasized, including regular voiding and avoidance of urine holding.

### 2.2. Diagnostic Assessment

A more severe breakthrough infection occurred in October 2025. During acute pyelonephritis, the white blood cell count was 25,610/µL with 90.2% neutrophils. After outpatient antibiotics, it was 18,850/µL with C-reactive protein of 15.612 mg/dL; urinalysis showed 1+ leukocyte esterase and 6–9 white blood cells per high-power field. Urine culture showed no growth may be due to urine collection post exposure to antibiotics.

**Figure 1:**
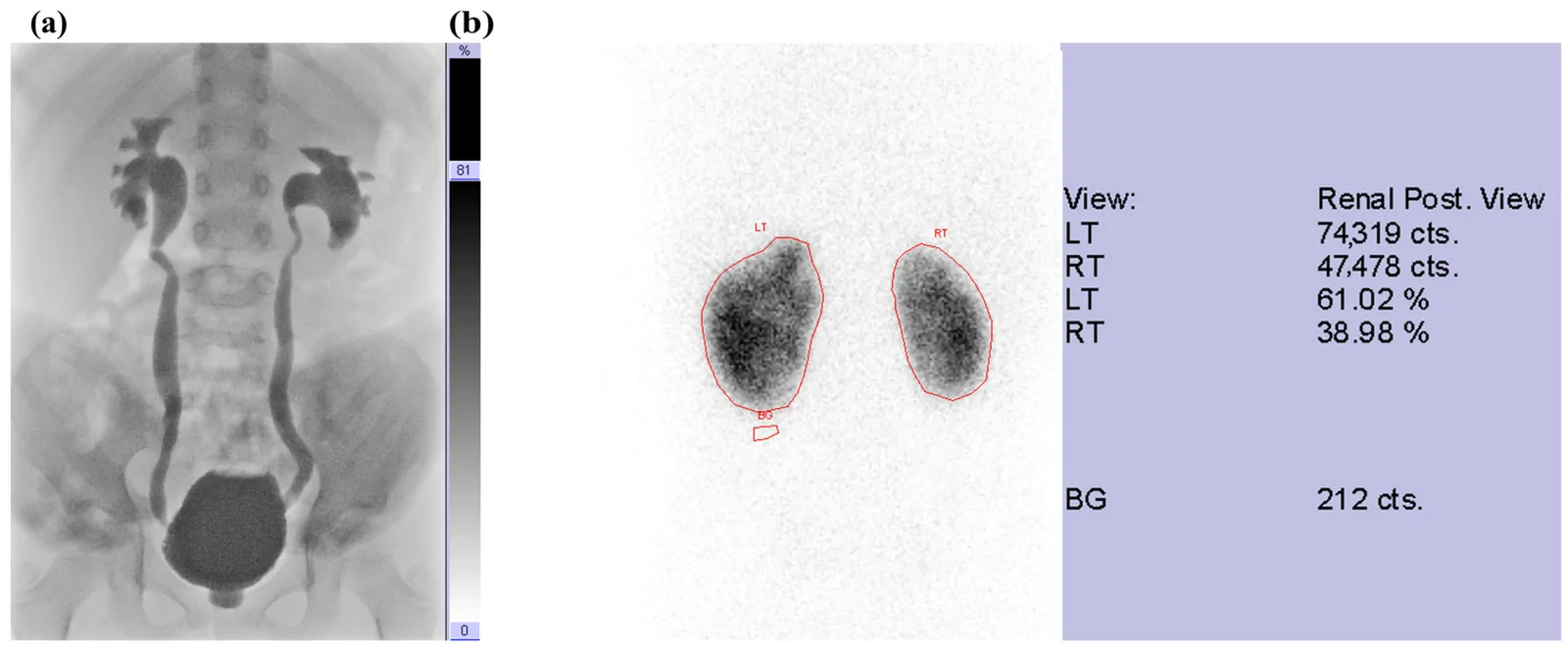
(**a**) High-grade bilateral vesicoureteral reflux (Right: Grade V, Left: Grade IV); (**b**) Preoperative technetium-99m dimercaptosuccinic acid (DMSA) renal scan demonstrated reduced relative cortical uptake by the right kidney (38.98%) compared with the left kidney (61.02%). Multiple areas of decreased tracer uptake were noted, consistent with cortical scarring from recurrent pyelonephritis. The red frame represented the left, right renal cortical area and background (BG).

Voiding cystourethrography (VCUG) confirmed persistent bilateral high-grade VUR: grade V on the right and grade IV on the left (Figure 1a). Technetium-99m dimercaptosuccinic acid (DMSA) renal scintigraphy demonstrated differential renal function of 38.98% in the right kidney and 61.02% in the left kidney. Multiple areas of decreased tracer uptake were present, consistent with cortical scarring from recurrent pyelonephritis (Figure 1b). Given the recurrent febrile/breakthrough UTIs despite two previous Deflux injections and persistent high-grade reflux, definitive surgical correction was chosen.

### 2.3. Therapeutic Intervention

**Figure 2:**
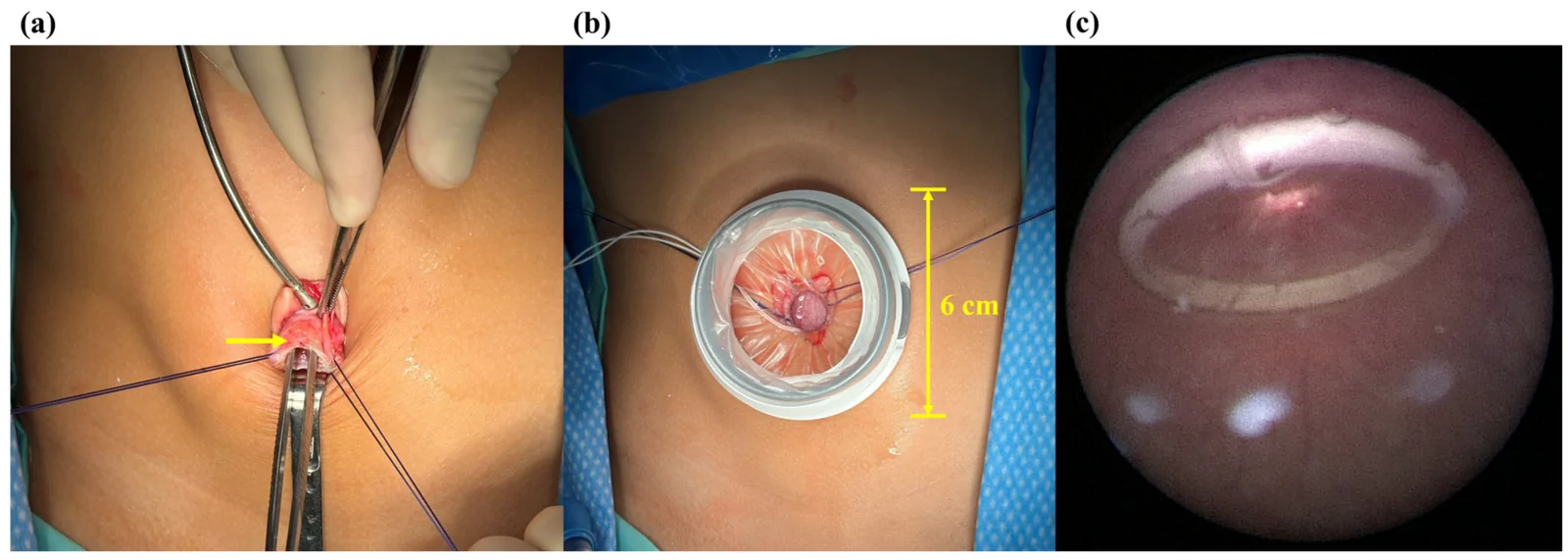
(**a**) A 2-cm transumbilical incision was created. Under direct cystoscopic guidance, two 2-0 Vicryl traction sutures were placed to facilitate bladder fixation. The arrow indicated the urinary bladder wall. (**b**) A single-port robotic access port with 6-cm external port diameter was then inserted directly into the bladder. (**c**) Confirm the position of port by cystoscopic visualization.

Under general anesthesia, the patient was placed in the lithotomy position. A 2-cm transumbilical incision was created. Under direct cystoscopic guidance, two 2-0 polyglactin 910 (Vicryl) traction sutures were placed to secure the bladder. A single-port robotic access port was inserted directly into the bladder, a pneumovesicoscopic working space was established, and the da Vinci SP system was docked (Figure 2). The expected bladder capacity (EBC) was estimated using two established age-based formulas. According to the International Children’s Continence Society (ICCS), EBC is calculated as 30 + [age (years) × 30] mL; therefore, the expected capacity for a 7-year-old child is approximately 240 mL [17]. Alternatively, using the formula proposed by Koff, bladder capacity (ounces) = age (years) + 2, the estimated capacity is 9 oz, corresponding to approximately 270 mL [18]. Thus, the expected bladder capacity for this 7-year-old patient was estimated to be approximately 240–270 mL. Unfortunately, there has been no established criteria or validated guideline defining the minimum bladder capacity required for robotic SP pneumovesicoscopic surgery. The measured distance from the umbilical incision to the target site at the bladder trigone exceeded 10 cm. We considered this umbilicus-to-trigone distance an important anatomical indicator of whether sufficient working length and intravesical space could be achieved for SP pneumovesicoscopic surgery. A da Vinci SP Small Access Port Kit (model 430073; Intuitive Surgical), designed for a 2.7–4.0-cm incision and featuring an approximately 6-cm external port diameter, was used.

**Figure 3:**
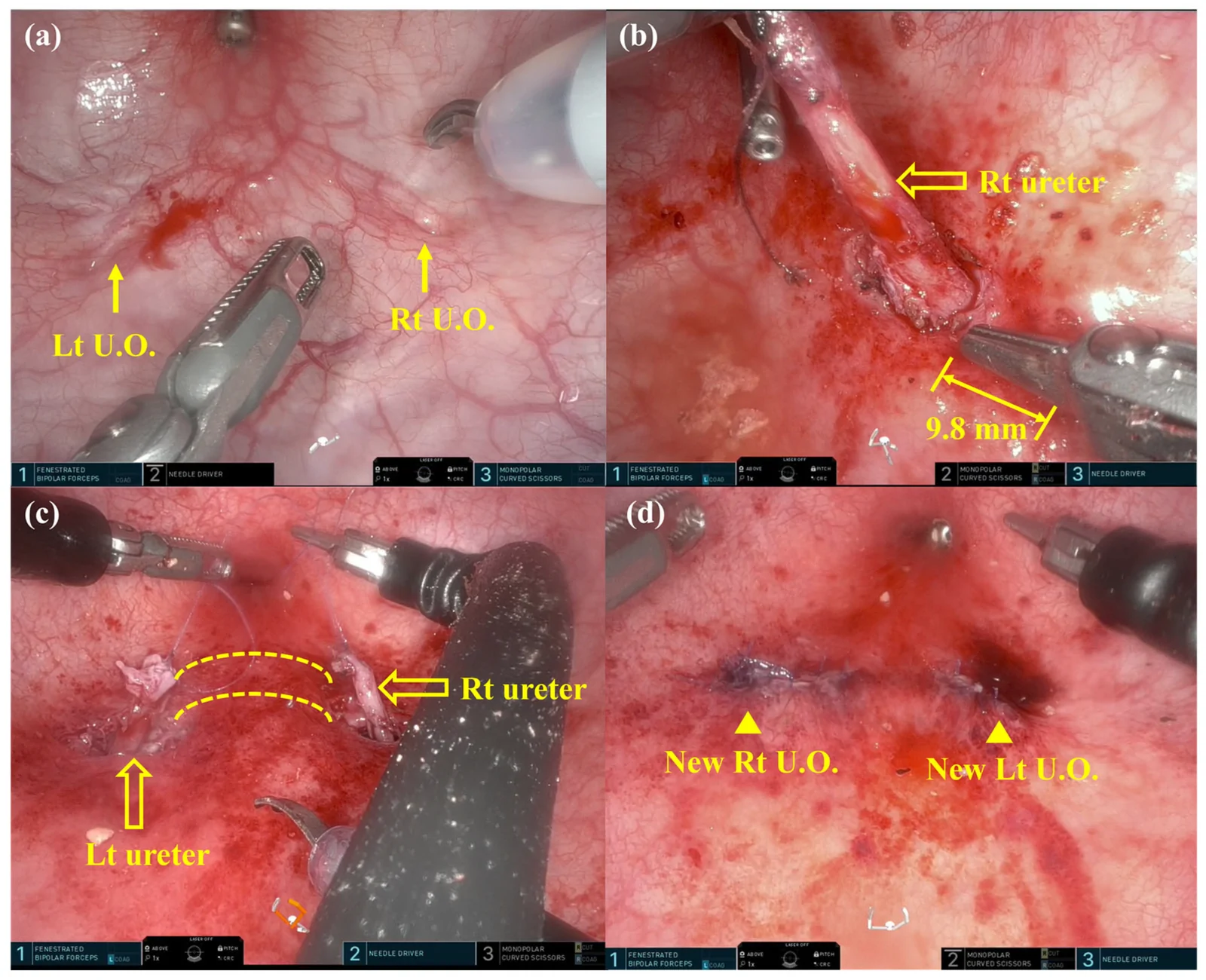
(**a**) Bilateral ureteral orifices (U.O.) were identified and marked with arrows. The monopolar curved scissors tip was measured 3 mm in width. (**b**) The hollow arrow indicated mobilized right ureter. The grip length of the needle driver was 9.8 mm. (**c**) The submucosal tunnels were created across the trigone (between the dashed lines). The hollow arrows indicated mobilized right and left ureters. (**d**) Ureters were repositioned via tunnels and secured with 5-0 Vicryl robotic suturing. The arrowheads represented the new ureteral orifices.

The bilateral ureteral orifices were identified (Figure 3a). Both ureters were mobilized, and residual dextranomer/hyaluronic acid copolymer was removed. Cross-trigonal submucosal tunnels were created according to the Cohen technique (Figure 3b,c). The length of submucosal tunnel was 2.5–3 cm with the both ureteral diameter as 3–4 mm. Each ureter was transposed into the contralateral tunnel. The creation of the new hiatus was performed with scissors and secured with interrupted 5–0 polyglactin 910 sutures using wristed robotic instruments (Figure 3d). The operation was completed without intraoperative complication or conversion.

The total anesthesia duration, operative duration and console duration were 375 min, 310 min and 250 min, respectively. There was minimal blood loss and no drainage tubes or Double J stents were placed. A 12 Fr silicone Foley was indwelled during the end of the procedure before closure of bladder. The pneumovesicum pressure was 12 mm-Hg. Cefazolin (750 mg intravenously every 8 h) was administered from postoperative day 0 through day 5.

### 2.4. Follow-Up and Outcomes

On postoperative day 2, one episode of emesis prompted radiography, which showed bowel dilatation and stool impaction. Symptoms resolved after a glycerin enema and brief bowel rest. She tolerated a regular diet by day 4; the Foley catheter was removed, and she was discharged on day 5. At approximately six months, she remained asymptomatic without dysuria, lower abdominal pain, flank pain nor fever. The most recent renal ultrasound (six months post Cohen) demonstrated normal renal echogenicity. Kidney measurements were: Right: 76.7 mm × 31.4 mm; Left: 90.0 mm × 41.2 mm. There was no hydronephrosis, calcification, focal lesion, mass, cyst, or fluid collection, and the urinary bladder had no structural abnormality or mucosal thickening. Urinalysis on August 19 was reassuring: RBC 0–2/HPF, WBC 0–5/HPF, with negative protein, occult blood, nitrite and leukocyte esterase; the urine was clear and yellow. The timeline of the reported case is listed as Table 1.

## 3. Discussion

Pneumovesicoscopic series report favorable success and shorter hospitalization than open surgery, although ureteral mobilization, submucosal tunneling, and suturing are demanding [1,2,3,4,5]. In this case, the articulating SP camera and wristed instruments enabled bilateral dissection, removal of prior injectable material, cross-trigonal tunneling, and suturing through one transumbilical access site, supporting technical feasibility.

The distinction between extravesical and intravesical robotic reimplantation is important. A systematic review of 22 studies involving 1362 children reported 92% overall success for robot-assisted extravesical reimplantation [8]. In contrast, intravesical robotic evidence remains limited; animal work and small multiport clinical reports demonstrated feasibility of transvesical Cohen reconstruction [9,10,11,12].

This case also differs from previous reduced-port reports. Pediatric da Vinci SP experience has mainly involved pyeloplasty, nephroureterectomy, and appendicovesicostomy [13]. Reduced-port pediatric ureteral reimplantation has used single-port-plus-one extravesical Lich-Gregoir configurations [14,15], whereas a 2026 report described pneumovesical Cohen reconstruction only in a juvenile pig using a 5G-controlled snake-arm system [16]. Thus, this appears to be the first clinical pure intravesical da Vinci SP Cohen reimplantation in a child, although novelty depends on the completeness of published evidence. The surgeon in charge had only limited experience of 10 SP procedures before this case, including pyloplasty, nephrectomy, hepaticojejunostomy, partial oophorectomy, pyeloplasty and intestinal duplication resection.

This report is limited by its single-case design, which precludes assessment of reproducibility, learning-curve effects, comparative effectiveness, cost-effectiveness, safety, and the durability of reflux resolution. Moreover, direct comparison with other established treatment modalities for pediatric vesicoureteral reflux, including open ureteral reimplantation, conventional pneumovesicoscopic techniques, multiport robotic surgery, clean intermittent catheterization, endoscopic injection therapy, and other bladder-directed treatments, was not feasible. Accordingly, the relative advantages and appropriate clinical role of single-port robotic-assisted pneumovesicoscopic Cohen ureteral reimplantation remain uncertain. Future studies should include larger multicenter cohorts with standardized long-term clinical, imaging, and renal functional follow-up, as well as comparative evaluation against established surgical and nonsurgical treatment strategies.

## 4. Conclusions

The pure intravesical da Vinci SP pneumovesicoscopic Cohen reimplantation was technically achievable for refractory bilateral high-grade VUR after failed endoscopic treatment. The procedure was completed without conversion or intraoperative complication, followed by discharge on postoperative day 5. Single-port articulation may facilitate complex bilateral intravesical reconstruction, even in a pediatric case.

## Figures and Tables

**Table 1:** Clinical timeline.

Date	Major Event	Key Findings	Treatment/Outcome	Clinical Significance
1 May 2019	First bilateral Deflux injection	Bilateral vesicoureteral reflux (VUR): Right IV/Left V; breakthrough urinary tract infection (UTI); mild bladder trabeculation	Deflux 1.0 mL each side	First endoscopic anti-reflux treatment; subsequent improvement to Right II/Left IV.
20 August 2019	Second bilateral Deflux injection	Residual VUR Right II/Left IV; loose ureteral orifices; migration of prior left Deflux	Repeat Deflux 1.0 mL each side	Persistent reflux despite first injection.
21–24 April 2023	Febrile UTI admission	Fever to 40 °C, CRP 12.3, pyuria	Cefepime → cefazolin + gentamicin; fever resolved	First documented later childhood hospitalization for breakthrough febrile UTI.
7–11 July 2024	Complicated UTI	WBC 15,220, CRP 6.0, pyuria; right mild hydronephrosis	Cefazolin + gentamicin; urine culture no growth	Second significant breakthrough UTI; imaging demonstrated right collecting-system dilatation.
10 July 2024	Uroflow/bladder scan	Qmax 29.2 mL/s, voided 178 mL, PVR 29 mL	Lifestyle/voiding modification	Good urine flow with only scant residual urine.
16–21 October 2025	Complicated UTI/suspected left acute pyelonephritis	Fever 40.8 °C, left flank pain, WBC up to 25,610, CRP 15.6	Multiple antibiotics; ultimately cefazolin + gentamicin; fever resolved	Severe recurrent breakthrough infection (febrile UTIs) despite two prior Deflux procedures.
Late Oct 2025	Renal/VUR reassessment	DMSA: Right 38.98%/Left 61.02%; on 31 October: Right Grade V/Left Grade IV	Decision for definitive surgery	Demonstrated reduced relative right renal function and recurrent high-grade bilateral VUR.
5 February 2026	Robotic bilateral ureteral reimplantation, Cohen method	Loose bilateral UOs, fibrosis around prior Deflux, chronic bladder inflammation	Definitive bilateral ureteral reimplantation	Major definitive surgical treatment after recurrent high-grade VUR and breakthrough UTIs.
5–10 February 2026	Post-operative recovery	Hematuria; transient vomiting/bowel dilatation with stool impaction	Conservative treatment; Foley removed on February 10; stable discharge	No major surgical complication; recovered sufficiently for discharge.
19 August 2026	Latest renal ultrasound and urinalysis	Small right kidney, no hydronephrosis; UA WBC 0–5, RBC 0–2, nitrite/LE negative	Continued follow-up	Reassuring post-operative urinary findings, while persistent right renal size asymmetry remains.

Abbreviations: CRP, C-reactive protein; WBC, white blood cell count; PVR, postvoid residual urine volume; DMSA, dimercaptosuccinic acid; UO, ureteral orifice; RBC, red blood cell count; UA, urinalysis.

## Data Availability

The data supporting the findings of this study are contained within the article. Owing to patient privacy and confidentiality, the underlying clinical records are not publicly available but are available from the corresponding author upon reasonable request.
